# Influence of Aging Technologies on the Volatile Profile Composition of Carignano *cv* Red Wines in Sardinia

**DOI:** 10.3390/foods14132290

**Published:** 2025-06-27

**Authors:** Giorgia Sarais, Mattia Casula, Francesco Corrias, Mariateresa Russo, Barbara Pinna, Francesca Argiolas, Mariano Murru, Alberto Angioni

**Affiliations:** 1 Department of Life and Environmental Science, University of Cagliari, University Campus of Monserrato, SS 554, 09042 Cagliari, Italy; gsarais@unica.it (G.S.); francesco.corrias@unica.it (F.C.); 2 Department of Agriculture, Mediterranean University of Reggio Calabria, 89124 Reggio Calabria, Italy; mariateresa.russo@unirc.it; 3 Argiolas Winery, 09040 Serdiana, Italy; barbara.pinna@argiolas.it (B.P.); francesca.argiolas@argiolas.it (F.A.); m.murru@argiolas.it (M.M.)

**Keywords:** aroma compounds, aging, stainless steel, concrete vat, plastic vat, barrique

## Abstract

Wine aroma is the result of the association of numerous volatile and non-volatile compounds belonging to the grapes, the fermentation, and aging process. During aging, wines complete their complex composition, and many aromas emerge. Therefore, aging represents a fundamental step to obtaining high-quality wines. Aromas belong directly to the odorless precursor in grapes or to the aging technology used. Analyses have been performed on wines obtained from the cv Carignano subjected to four aging technologies: stainless-steel tank, plastic vat, concrete vat, and oak barrel. GC/FID and GC/MS analysis allowed the identification of 78 significant compounds belonging to eight different chemical classes. Volatile composition in the various containers was assessed at two levels: chemical classes and individual compounds. At 12 months, plastic vats had the highest increase in the total VOC concentration (*p* < 0.05), followed by concrete and stainless steel. In contrast, oak barrels showed a decrease, although the difference was not statistically significant (*p* > 0.05). Unsupervised principal component analysis (PCA) demonstrated that the container exerts a more substantial influence at 6 months, while at 12 months, the samples were categorized irrespective of the container. In the loading plot, several esters, acids, lactones, and aldehydes showed negative loadings on PC1 (associated with time), indicating a correlation with the 12-month collection time.

## 1. Introduction

The overall aroma of wine results from a complex association of many different volatile organic compounds (VOCs) originating from grapes during the fermentative process and storage, depending on the aging conditions [1,2,3]. Therefore, it is not related to one single compound but to a synergistic and additive effect of many compounds belonging to different chemical classes, such as terpenes, norisoprenoids, thiol derivatives, lactones, and precursors of alcohols and aldehydes, mainly conjugated with sugars and amino acids. To date, more than 1000 compounds have been described in wine, with variable concentrations that can participate directly in the overall aroma or indirectly by enhancing the flavor characteristics of other compounds in the bouquet [4,5,6,7].

The fermentative step has been considered the most important for generating volatile compounds due to high yeast activity [5]. However, aging represents a fundamental step for high-quality wines; during this period, many aromas emerge from odorless precursors present in grapes [6], improving the wine’s sensory characteristics and complexity, from oxidation or reduction reactions depending on the aging technology employed [3,4,5,6].

The word aging includes two distinct phases of wine life with different purposes; the first is made in bulk and pertains to the maturation of the wine, and the second, in the bottle, consists of refinement. Bulk maturation can be performed with inert containers like stainless-steel tanks, plastic vats that do not interact with the wine, and concrete vats or oak barrels (like French “barriques”), which can actively or passively interact with the wine. Stainless-steel is the most popular type of tank, as it allows the precise control of the temperature and is easy to sanitize; the wines are stored in an anaerobic environment, so it is a suspended chemical situation in which changes occur slowly. Stainless-steel tanks preserve wine’s fresh, fruity characteristics, and create a reductive environment, facilitating some chemical reactions and avoiding others [8].

Plastic vats are used more in small-scale wineries for their low cost and ease of use. Wines aged in plastic tend to be soft, velvety, and smooth [9,10]. Concrete vats have been recently rediscovered; they showed better thermic inertia and micro-oxygenation of the wine comparable to oak barrels, but not imparting oak aromas and flavors. Moreover, concrete vats produce wines with a fresh and clean aromatic profile, like stainless steel, but with a palate sensation that is less harsh and toned [11].

Oak barrels have a wide range of variables related to the type of oak, the process used for the preparation of the barrel, the dimensions of the barrel, the time of aging in the barrel, and how long the barrel has been used. The influence of some of these factors (mainly geographical origin, toasting degree, and size) on wines’ phenolic and volatile composition has been widely studied. Oak is naturally porous, allowing a continuous diffusion of oxygen, which modulates the behavior of many reactions involving volatile and non-volatile compounds, influencing the final aroma of wine [12,13,14]. Moreover, when the wines are stored in the barrel, some evaporation inevitably occurs, and a part of the wine could be lost via evaporation; this natural process results in an increased concentration of the wine’s aromatic and flavor compounds.

Oak also contains essential volatile compounds that significantly impact wine aroma. Oak barrel aging is unsuitable for delicate wines because oxygen could oxidize the wine, and the oak-derived components related to the oak origin could suppress its sensory characteristics [14].

The chemical analysis of wine aroma is complex due to the high number of compounds and different chemical classes with volatile and non-volatile characteristics. Therefore, many different analytical approaches can be applied, leading to different conclusions. The most used methodologies are represented by gas and liquid chromatography coupled with mass spectrometry (GC-MS, LC-MS), or other less discriminant detectors [15,16]. Gas chromatography–olfactometry (GC-O) uses the human nose as a detector to obtain odor information from volatile compounds separated into a chromatographic column [17,18]. GC associated with an electronic tongue based on voltametric sensors and elaboration through principal component analysis (PCA) can discriminate between wines aged in oak barrels and stainless-steel tanks in contact with oak chips [19].

Sample preparation can significantly affect the resulting determination of aroma compounds. Liquid–liquid extractions, direct analysis, and static or dynamic headspace can be used before chromatographic determination to select and concentrate volatiles and aromas; however, each has bottlenecks. Due to the limits of each analytical technique used, researchers focused on the identification of compounds generating characteristic hints, such as green pepper notes in Cabernet related to 2-methoxy-3-isobutylpyrazine, curry notes in Porto, and sweet Grenache fortified wines attributable to sotolon [20,21], and exotic fruit notes in Cabernet Sauvignon and Merlot wines attributable to thiol compounds [22].

Carignano wine has recently received great appreciation among producers and consumers for its full-bodied and aromatic characteristics. The origin of the vine is unclear; some trace it back to the Phoenicians, others to the Aragonese, hypothesizing a derivation from Spanish vines. Carignano is a vigorous vine with abundant and constant production, poorly resistant to cryptogams and powdery mildew. On the other hand, it has good resistance to salty winds and cold spring temperatures. Therefore, a vine can grow well along the coasts in sandy soils. These characteristics have allowed the use of ungrafted vines since these environmental conditions are not ideal for spreading phylloxera (*Daktulosphaera vitifoliae*) [23,24].

Carignano is grown in the traditional “South-Western” area of Sardinia, which also includes the island of Sant’Antioco and San Pietro, reaching 70% of the varieties in this area and producing a well-structured wine with high alcoholic content. Despite its limited diffusion, Carignano is undoubtedly one of Sardinian oenology’s most interesting and prestigious grape varieties, with an intense ruby red color, vinous perfume, and dry, spicy, and harmonious taste [25,26]. The wine-making processes allow for aging between three months and a year before bottling. Although the importance of this grape variety is acknowledged, no studies have been found concerning the influence of aging technology on the aroma profile. Therefore, this paper presents, for the first time, a comparative study of the evolution of the volatile fraction composition of wines of the *Carignano cultivar* subjected to aging for 12 months in four different containers: stainless-steel, plastic, concrete, and oak barrels.

Through the volatile fraction characterization, this paper intends to understand the effect of different aging techniques on the chemical composition of the red wine Carignano. Analyses were made by GC-FID and GC-MS after volatile concentrations using an already validated extraction and partition method [27], and statistical comparison was carried out using principal component analysis (PCA) and two-way analysis of variance (ANOVA), followed by Tukey’s test.

## 2. Materials and Methods

### 2.1. Reagents, Samples, and Standards

Methanol (HPLC grade) and absolute ethanol (ACS quality) were supplied by Merck (Milan, Italy). Anhydrous magnesium sulfate (MgSO_4_), with a particle size of 45–75 μm, and tartaric acid were ACS-ISO quality (Fluka, Milan, Italy). Before use, bidistilled water was obtained from a Milli-Q purification system (Millipore, Milan, Italy). The chemical standards (pure reference compounds, 97–99%) were purchased from Sigma-Aldrich (Milan, Italy) (Table S1). Wine samples were obtained from the cv “Carignano” from a unique vineyard subjected to standard agriculture procedure (GAP) with a plant spacing of 2 × 3 m and were kindly supplied by Argiolas Winery (Serdiana, Cagliari).

Stock standard solutions (5000 mg/L) of the active ingredients were prepared in methanol and stored in the dark at a low temperature (−20 °C); intermediate spiking solutions were prepared by diluting known aliquots of stock standard solutions with methanol. Working standard solutions were prepared from the intermediate solution daily before analysis.

### 2.2. Aging Conditions

The Carignano wine used for the trials was obtained from standard winery operations. Briefly, the grapes were collected at ≥ 26 °Bx (Brix), berries were separated from the stems, and the must was added with 2 g/hL of sulfur dioxide for a total value of about 30 mg/L of free and 90 mg/L of total sulfur dioxide; the addition was made with powdered metabisulfite and fermented in stainless-steel tanks activated with previously activated dry yeast (500 mg/kg of *Saccharomyces cerevisiae*/grapes). At the end of the fermentation, wines were subjected to pumping over, racking (lees and wine), clarification, and malolactic fermentation (4–5 days). Thereafter, the bulk wine was divided into four different aging containers for storage and aging: 200 L stainless-steel tanks (SS) and plastic vat (P), 2500 L concrete vat (C), and 225 L high-quality new French oak (*Quercus petraea*) barrels (OB) subjected to light toasting. Bulk wine was added to the oak barrels during the experiment to restore the initial volume and avoid concentration and evaporation of the wine. The containers were maintained at 15–18 °C and an average relative humidity of 60–70%. Samples were collected on the bulk mass before aging (0_m), in the containers (T) after six months (T_6m), and 12 months (T_12m). Each experiment was carried out in triplicate.

### 2.3. Sample Preparation and Analysis

Samples were prepared and analyzed according to Angioni et al. [27]. An amount of 5 mL of wine was poured into a screw-capped centrifuge tube (15 mL) with 3.5 g of anhydrous MgSO_4_. The obtained mixture was mixed using a stainless-steel micro spatula to achieve a homogeneous suspension. The tube was centrifuged for 3 min at 1.957 relative centrifugal force (RCF) and 14 °C; following this operation, two phases were obtained: an upper liquid phase containing ethanol, glycerol, and the aromatic fraction, and a lower phase comprising water and MgSO_4_. The upper phase was withdrawn, diluted (1:1, *v*/*v*) with methanol, and filtered with an Acrodisc CR 4 mm Syringe Filter 0.45 µm PTFE membrane (Waters, Milan, Italy). Samples were injected without any clean-up step in the GC-FID and GC-MS systems.

### 2.4. Analytical Instrumentation

Analyses were carried out using two analytical platforms: gas chromatograph Trace (Thermo Finningan, Rodano, Milan, Italy) coupled to a flame ionization detector (GC-FID), an autosampler AS 800, and a split–splitless injector, used for the quali-quantitative analysis, and a gas chromatograph Trace GC ultra (Thermo Finnigan, Waltham, 02454 MA, USA), coupled to a single quadrupole mass selective detector (GC-MSDSQ), connected with the software Xcalibur 4.1, for the identification and characterization of the compounds in the aroma fraction.

The capillary column was a CP-WAX 57CB Varian (60 m long, 0.25 mm, i.d., and 0.25 μm thickness: Varian Inc., Palo Alto, CA, USA). The carrier gas was He at 1 mL/min, and the injection volume was 1 μL (FID, split mode 1:20; MSDSQ splitless 60 s). The injector was set at 200 °C, whereas the detectors were set at 280 °C (FID) and 200 °C (MSDSQ). The oven was programmed as follows: 50 °C (1 min.), till 220 °C (4 °C/min) and held for 7.5 min. The FID make-up gas was N_2_ at 80 Kpa.

MSDSQ acquisition was carried out at 70 eV in EI positive ionization mode, and mass spectra were recorded in full scan mode, with the mass-to-charge ratio (*m*/*z*) ranging from 65 to 450 u. Single-compound identification was carried out by comparison of their relative retention times and mass fragmentation with those of computer matching against the commercial library (NIST 17 EI MS Library 2019) and homemade library mass spectra from pure reference substances and previously identified compounds in wine.

### 2.5. Odor Activity Values (OAVs)

The overall aroma contribution of wine was evaluated using the Odor Activity Value (OAV). The OAV is calculated by dividing the concentration of volatile compounds by the odor threshold (OTH). Volatile compounds with an OAV greater than 1 are aromatically active and play an essential role in developing the wine’s aromatic profile. When the OAV is close to 1, it suggests that the volatile compound is present at a concentration similar to its OTH. In this case, the compound may have a subtle influence on the aroma. If the OAV is less than 1, the volatile compound is present at a concentration below its odor threshold. Such compounds are considered non-odor-active and are unlikely to contribute significantly to the aroma [28].

### 2.6. Statistical Analysis

The effect of time was evaluated by performing multiple unpaired t-tests comparing the 0_m group with the samples collected at 6 and 12 months.

Furthermore, to evaluate the influence of the container type, time, and their mutual interaction, a two-way analysis of variance (ANOVA) followed by Tukey’s test was performed in each chemical class (e.g., aldehydes, acids, alcohols, etc.), with time and container type as independent factors. All statistical investigations were performed by GraphPad Prism software (version 8.3.0, Dotmatics, Boston, Massachusetts). For each analysis, the *p*-values and the effect size (η^2^—eta squared) were calculated [29].

GC-MS datasets were imported into SIMCA 13 (Umetrics AB, Umea, Sweden) for processing principal component analysis (PCA). Additionally, R^2^ (the determination coefficient) and Q^2^ (the cross-validated correlation coefficient) were used as measures for the robustness of a pattern recognition model [30].

## 3. Results and Discussion

Wine aroma is a complex mixture of volatile and non-volatile compounds perceived in the nose and the mouth with concentrations ranging from mg/L to ng/L originating from the grape (primary aromas) during yeast fermentation, aging, or recovering from the containers [5,31].

The process of aging wine is a fundamental step toward obtaining high-quality wine. During this period, wine acquires aromatic complexity due to biochemical reactions such as esterification, hydrolysis, redox reactions, spontaneous clarification, CO_2_ elimination, and slow and continuous oxygen diffusion [2,32].

Carignano wine was obtained from controlled fermentation in a single big container using standard winery operations and divided into 12 containers. The containers were stainless-steel, plastic, concrete vats, and oak barrels; the wine was aged for 12 months, with an intermediate sampling at 6 months.

The FID gas chromatographic analysis revealed the presence of 20 compounds and could not differentiate volatiles among the four containers (Figure S1). Therefore, GC-MS analysis was carried out to better characterize the volatile fraction and highlight the differences among the aging technologies. The analytical method highlighted the presence of 78 compounds, of which 14 could not be assigned a precise structure, only the chemical family (Table S1).

During wine maturation, the type of container (stainless-steel, concrete, plastic, or oak) and the aging time (6 or 12 months) may cause significant changes in the composition of volatile compounds [33,34]. These variations affect Carignano’s aromatic profile by modifying the compounds in the various chemical classes.

**Figure 1:**
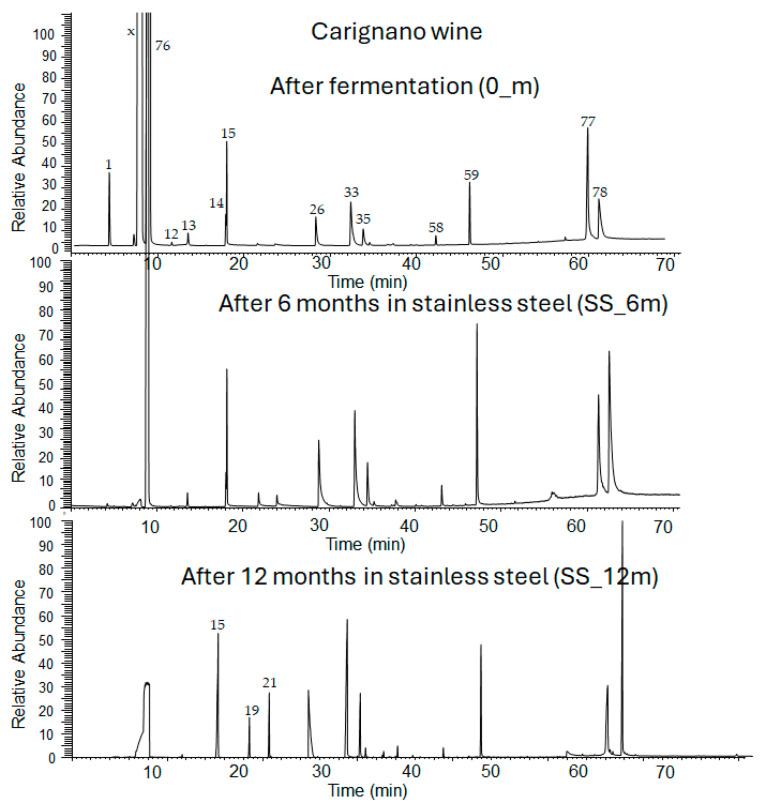
GC-MS chromatogram of Carignano wine after fermentation and aging in stainless-steel (SS) tanks. X is methanol used as a solvent; the numbers on the peaks refer to the compounds in Table S1.

The detected compounds changed in number, type, and amount at the different collecting times and aging technologies (Figure 1). After the fermentation step, 31 compounds were detected, whereas after six months, between 34 and 38, and at 12 months, between 62 and 66.

**Figure 2:**
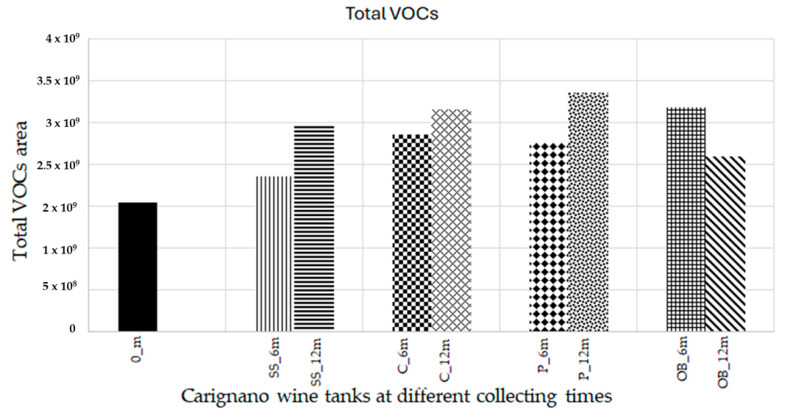
Total area of VOCs at 0, 6, and 12 months of aging without the amount of ethanol and glycerol.

**Figure 3:**
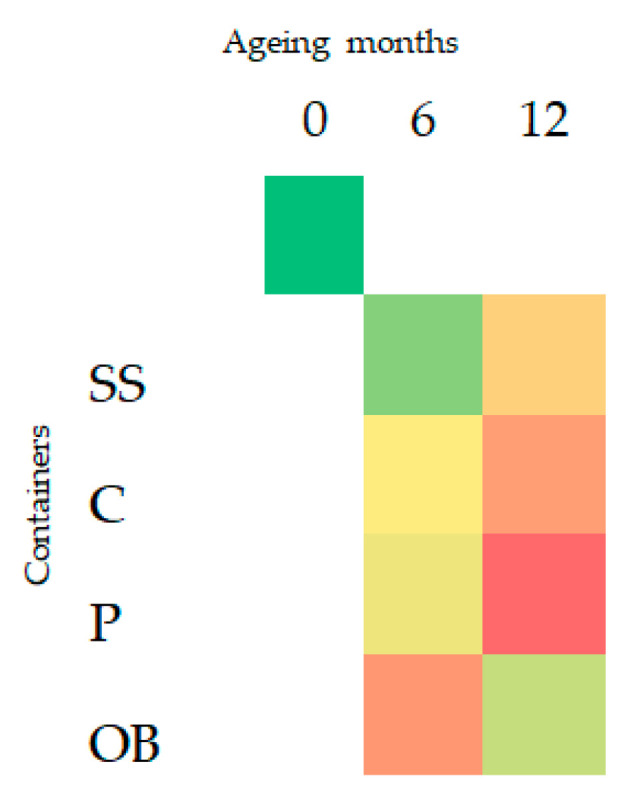
Heatmap of total VOCs at different aging times (0, 6, 12 months) and in the different containers: stainless-steel (SS), concrete (C), plastic (P), oak barrel (OB). Color from green to red had increased concentrations.

The total concentration of VOCs (volatile organic compounds) at different collecting times showed an increase in all containers after six months. At 12 months, plastic vats had the highest increase (*p <* 0.05), followed by concrete and stainless steel, whereas oak barrels had a decrease in the total concentration of VOCs (Figure 2 and Figure 3).

The volatile compounds detected could be classified into eight chemical classes. The most represented was the alcohol fraction, accounting for on average in all samples 90.7% ± 8.6%, mostly related to the presence of ethanol and glycerol.

Since ethanol and glycerol had a big impact on the volatile fraction, the analysis of the individual chemical classes was elaborated without these two compounds. Once again, the alcohol group was the most abundant among the four aged wines, ranging from 61.6 to 76.4% of the total VOCs, followed by acids (12.4 to 22.0%) and esters (6.6 to 9.4%). The total alcohol increased in the first 6 months but decreased after 12 months for *p* < 0.0001 (Table 1 and Table S2A).

The decrease in the alcohol group in all containers between 6 and 12 months was related to the increase in the ester group, with aroma compounds associated with fruity profiles (Table S3) [35]. These data agree with previous studies on Cabernet Sauvignon aged in oak barrels regardless of the type of the barrel [36].

Aldehydes statistically differ over time (Table S2A) for *p* ranging from 0.04 to 0.0004 at 6 months, whereas for *p* < 0.0001 at 12 months. The containers T_6m vs. T_12m showed significant differences for *p* < 0.0001, except for concrete (*p* < 0.0097) and oak barrels (*p* < 0.0021) (Table S2B).

Ketones showed an increase in T_12m vs. 0m in all containers; moreover, an increase was also registered from T_6m and T_12m, with OB and C wines exhibiting a lower increase. All containers were statistically different for *p* < 0.0001. At six months, no statistical differences were evidenced among containers (Table S2B).

At 6 months, esters showed differences among P_6m, SS_6m, and OB_6m. After an initial decline, they increased at 12 months for *p* < 0.0001. OB exhibited the highest concentrations, suggesting a positive effect of this material on ester formation during aging. The levels of ester compounds increased according to the following trend: oak > plastics = concrete > stainless steel (Table 1).

The results of the multiple t-test comparisons and the two-way ANOVA showed significant effects of the time and container type on the concentration of the different chemical classes analyzed, as well as significant interactions between these two factors (Table S2A,B). The effect size index, calculated as eta squared (η^2^), was used to estimate the relative importance of each factor on the total observed variability (Table S3). Time emerged as the primary factor driving the evolution of the different chemical classes, accounting for the main variance in all chemical classes (η^2^ 0.79 in aldehydes, 0.74 in acids and in lactones). The effect of time was also particularly pronounced for ketones (η^2^ = 0.47) and esters (η^2^ = 0.46). Although the *p*-values showed statistically significant differences in all combinations, the effect size for the containers and the interaction time x containers were all below 0.27, indicating a small effect of the parameter studied. This suggests that the type of container contributes to chemical differences, but to a lesser extent than the storage time.

**Figure 4:**
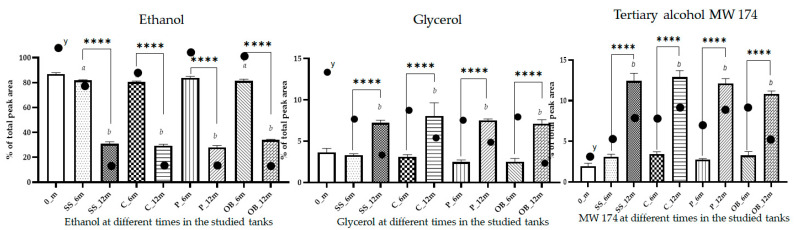
Column plots showing the relative abundance (%) of ethanol, glycerol, and MW174 in Carignano wine samples across different aging stages and storage container types. Significance differences among columns: **** *p* < 0.0001; different letters denote statistically significant differences over time: (*a*) 0_m vs. T_6m, (*b*) 0_m vs. T_12m (*p* < 0.05). ^y^ absolute abundance in the analytical extract.

The analysis of the single compounds highlighted that 0_m and T_6m wines were characterized by ethanol, glycerol, and a diol with MW 174 and chemical formula C_10_H_22_O_2_, representing on average 88.8% ± 2.4% of the total volatiles; whereas, after 12 months (T_12m), these compounds accounted for 50.9% ± 4.0% (Figure 4). Ethanol was the main volatile detected in all experiments, and the alcohol degree of the wine remained constant during the experiment at 14%. The apparent decrease in ethanol from 6 to 12 months in all containers was ascribed to the emergence of new compounds, which almost doubled after 12 months, and the increase in some of them in the overall composition of the volatile fraction (Table S1). It was demonstrated by the evidence that whilst there may not be a marked change in the overall ethanol content, its perceived sweetness and the way it interacts with other elements of the wine can be modified by reactions such as esterification [34,36,37,38]. The absolute concentration of ethanol in the volatile fraction showed values ranging from 461,2 to 630,5 mg/L with an average value of 555.7 ± 9.3% (mg/L ± RSD%), confirming that the decrease in percentage was only apparent due to the number of volatiles present in aged wine (Figure S2).

The second most represented compound was a diol with MW 174 and chemical formula C_10_H_22_O_2_, with values ranging from 1.96 ± 18.02% to 3.43 ± 8.42% (mean ± RSD%) in the first 6 months and almost 12% after 12 months of aging (Table S1). The absolute concentration increased during storage, except in oak, where a net decrease was registered. Glycerol was the third most common chemical compound in wines. Glycerol is a non-volatile important by-product of alcoholic fermentation, which has no aromatic properties, but contributes significantly to wine appreciation by providing sweetness and fullness [37]. Usually, the glycerol concentration in wines is around 5 g/L, but the amounts can be as high as 15–20 g/L and depend upon the fermentation conditions, especially the level of sulfur dioxide [39]. The data acquired in these trials showed that the aging time and vessels influenced the glycerol weight in the overall matrix volatiles’ percentual composition. It decreased from 3.66 ± 13.2% (0_m) to 3.30 ± 4.7% in SS_6m, 2.47 ± 11.6% in P_6m, 3.11 ± 8.3% in C_6m, and 2.55 ± 15.1% in OB_6m, whereas its percentage increased after 12 months to a level of almost 7–8% in all containers, with the higher values in concrete (Figure 4). However, during aging, glycerol undergoes a reaction of condensation with acetaldehyde and other compounds, decreasing over time. The apparent glycerol% increase may be attributed to the decrease in the importance of the ethanol% amounts; the absolute concentration of glycerol confirmed the decrease in the 12-month samples (Figure S2).

The main compounds (not considering ethanol, glycerol, and MW174) in the 0_m and T_6m wines were represented by (R,R, and R,S)-2,3-butanediol, isoamyl and N-amyl alcohols, and benzenethanol, followed by acetic acid and acetic acid ethyl ester.

After 12 months, there was an increase in iso-amyl alcohol, 2-butanone 3-hydroxy, lactic acid and lactic acid ethylester, and (R,R)-2,3-butanediol, the disappearance of N-amyl alcohol, ethyl, and methyl esters of acetic acid, and a decrease in glyceraldehyde and isobutyl alcohol. On the contrary, lactone and new ester compounds were occurring in all aging containers (Table S1).

**Figure 5:**
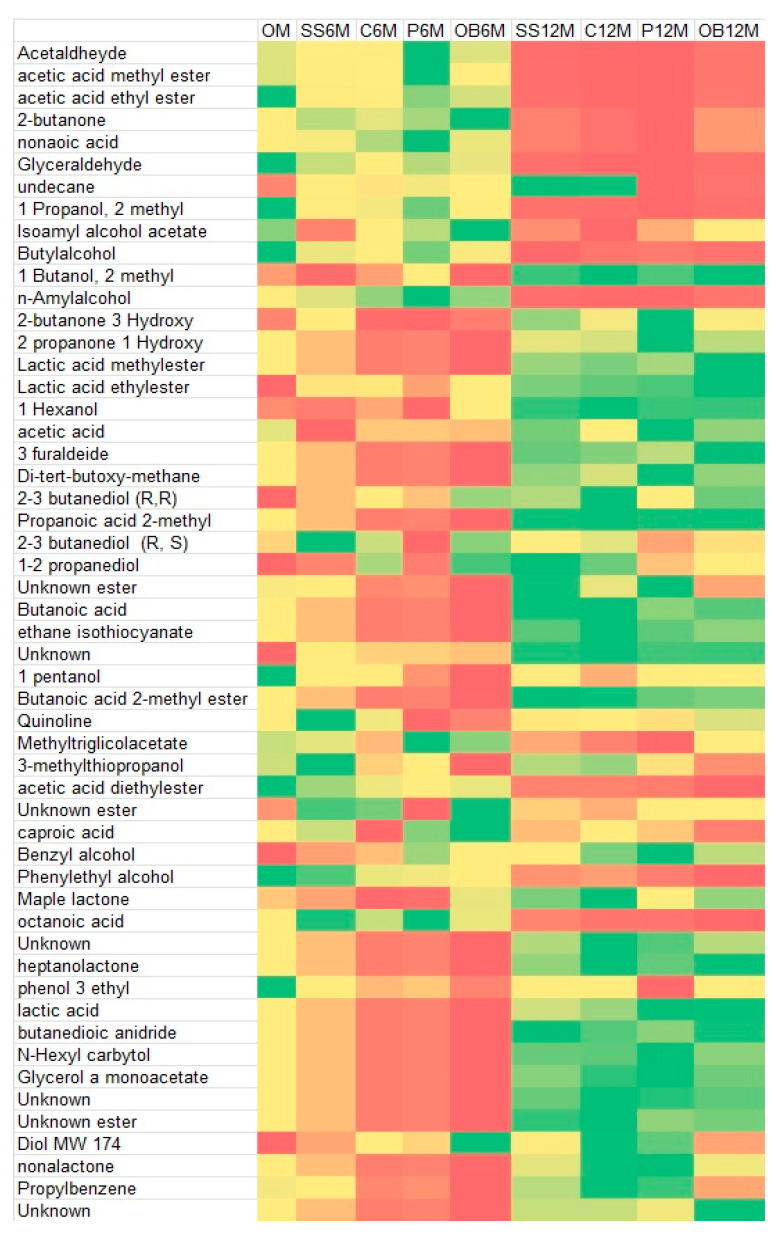
Heatmap of main VOCs at different aging times (0, 6, 12 months) and in the different containers: stainless-steel (SS), concrete (C), plastic (P), oak barrel (OB). The green zone represents more concentrated compounds, whereas the red zone represents less concentrated.

The heatmap of the main single-chemical compounds showed a different distribution of volatiles, mainly influenced by the time of aging and less by the container. After 12 months, a higher abundance of complex compounds, like esters and lactones, can be noted (Figure 5).

**Figure 6:**
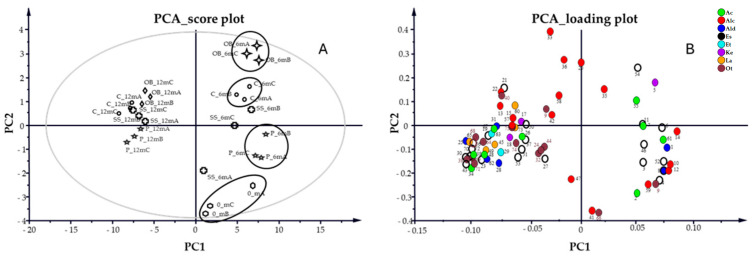
PCA score plot (**A**) and loading plot (**B**) of wine aging. In the score plot, the confidence interval is defined by Hotelling’s T2 ellipse (95% confidence interval).

Principal component analysis (PCA) using three principal components (PCs = 3) was applied to better discriminate among trials (Figure 6). The individual R^2^ and Q^2^ values for each component were as follows: PC1—R^2^ = 0.61, Q^2^ = 0.57; PC2—R^2^ = 0.70, Q^2^ = 0.63; and PC3—R^2^ = 0.77, Q^2^ = 0.65. All available variables were initially considered; however, ethanol, glycerol, and the MW 174 diol were excluded from the final PCA model as they were the most abundant components and could influence the model by masking small variations within the dataset. The score plot (Figure 6A) showed that the wine samples clustered primarily according to aging time, rather than other factors. The first two principal components of the PCA model resolved 70.3% of the total data variance. Samples aged 6 and 12 months make two distinct clusters along the first principal component, representing the direction of maximum variance (61% of explained variance). Non-aged samples (0_m) were separated along the second principal component (PC2, 9.3% of explained variance). In the 6m aged samples, some dispersion was observed along PC2, allowing the identification of four subgroups associated with different container types. In contrast, the 12m aged samples appeared more tightly clustered, suggesting that a more extended aging period reduces the influence of the container on the overall volatile compound profile (Figure 6A).

Analysis of the corresponding loading plot allows the identification of the variables that mainly influenced the samples’ distribution. Esters (Es), acids (Ac), lactones (Lac), and some aldehydes (Ald) showed a stronger correlation with the samples aged for 12 months, suggesting an increase in their concentration during the aging time. The PCA analysis of single compounds showed that 2-butanone (5), an unknown ester (54), and caproic acid (55) were the most important variables affecting the OB_6m and C_6m shift along PC2, whereas 1-pentanol (41) and phenol 3-ethyl (66) were related to the 0_m samples (Figure 6B).

The P_6m samples were better characterized by the group of esters acetic acid diethyl ester (2), acetic acid methyl ester (3), acetic acid ethyl ester (4), acetic acid vinyl ester (6), isoamyl alcohol acetate (11), and methyl-triglycol-acetate (46), together with acetaldehyde (1), glycolaldehyde (8), and the alcohols 1-propanol-2-methyl (10), butyl alcohol (12), and n-amyl alcohol (14) (Figure 6B). Regarding the SS_6m samples, they were spread on the right space of the score plot without being influenced by single compounds.

At the end of the aging period (12 months), samples were grouped regardless of the container, even if minimal clusters along PC2 were evidenced, with OB_12m in the left positive space and P_12m in the left negative space of the plot (Figure 6A). The compounds influencing the OB_12m samples more were 1-butanol, 2-methyl (13), lactic acid ethylester (21), 1-hexanol (22), an uncharacterized compound (40), and maple lactone (60). P_12m were influenced by 1,3 Butadiene 2,3-dimethyl (24), 4 methyl 2 penthyl acetate (27), a hydrocarbon (32), and quinoline (44) (Figure 6B).

The asymmetric distribution of compounds in the loading plot, with 29% located on the right side and 71% on the left side of the multivariate space, determined the separation between the T_6m and T_12m samples observed in the score plot.

Compounds 54, 5, 55, and 35 showed positive loadings on both PC1 and PC2, and were associated with the OB_6m and C_6m samples.

On the contrary, five esters (3, 4, 6, 46, and 52), five alcohols (10, 12, 14, 41, and 59), two aldehydes (1 and 8), and undecane (9) exhibited negative loadings along PC2 and were associated with the P_6m samples.

In the left space, eight alcohols (13, 15, 22, 33, 36, 42, and 58), maple lactone (60), compounds 40 and 9, and 2-butanone-3-hydroxy (17) showed negative loadings on PC1 and positive loadings on PC2 (Figure 6B). All other compounds showed a negative correlation in both PC1 and PC2.

**Figure 7:**
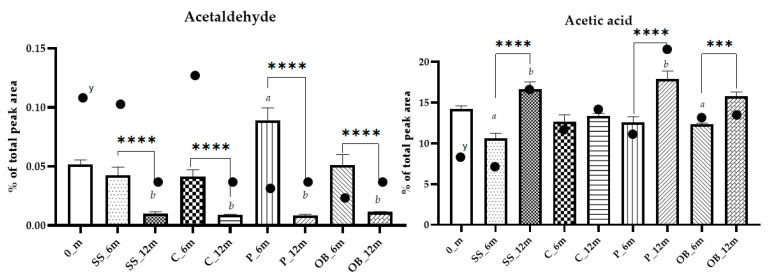
Column plots showing the abundance of acetaldehyde and acetic acid in Carignano wine samples aged for 6 or 12 months in different containers. Differences were tested using Tukey’s post hoc test. *** denotes *p* < 0.001 **** denotes *p* < 0.0001. Different letters indicate statistically significant differences (*p* < 0.05) between aging times within each container type. ^y^ absolute abundance in the analytical extract.

Aldehydes such as acetaldehyde (1) are mainly derived from fermentation but can be formed from ethanol by oxidation during aging [5,38]. Acetaldehyde originates principally in fermentation, being an intermediate in ethanol and glycerol production in the glycolysis pathway, depending on the availability of oxygen [40]. Its levels remained stable in SS_6m, C_6m, and OB_6m but increased in P_6m, whereas the levels were below 0.01% in all the samples after 12 months (Figure 7). Acetaldehyde undergoes several reactions during aging, combining with flavanol ethyl-linked oligomers to form a complex polymeric-type structure. Moreover, it can be formed from ethanol by oxidation in containers with high oxygen transfer [41]. In addition, glyceraldehyde showed a decrease during aging in all containers, also confirming the absence of oxidation reactions in the OB probably related to the type of wood, and confirming the assumption that antioxidant compounds in wine can deactivate acetaldehyde [42].

After 12 months, the total organic acid increased for *p* < 0.05, in SS_12m, P_12m, and OB_12m, whereas C_12m remained stable (Table 1 and Table S2A). Among acids, acetic and lactic acid were the most abundant and showed clear increases over time. Acetic acid showed the most pronounced increase, rising from initial values around ~1.1 ± 8.4% to ~9.3 ± 6.2%, with the highest concentrations observed in plastic vats and oak barrels (Figure 7).

As a porous material, oak allows for slight but continuous oxygenation of the product. This slow oxygen exchange promotes several chemical reactions, including oxidation [38]. Depending on the type of concrete, some gaseous exchanges could be assumed, whereas stainless-steel tanks are described as a highly ‘inert’ material, and are not responsible for any exogenous compounds that positively impact the sensory properties [43]. In addition, plastic vats’ gaseous exchanges are related to the density of the material and the size of the maturation vessel [44].

The oak barrels used in this study were of French oak. This wood is characterized by a fine and dense grain with a compact arrangement of the wood fibers, therefore allowing a controlled and gradual transfer of oxygen to the wine. French oak barrels do not *over-oxidize* the wine and show a low concentration of lactones. Moreover, light toasting allows only a minimum release of phenolic compounds from the wood, enhancing the wine’s fruit, with a slight contribution of spicy and smoky notes. Wine aged in French oak barrels requires more time in contact with the wood to develop its characteristics, as the release of oxygen and aromatic compounds is slower and more controlled.

The concrete vats had an internal epoxy resin to coat the walls, which reduced the gaseous exchange with the ambient. The plastic vats had a medium/high density allowing small gaseous exchanges. Moreover, the temperature (15–18 °C) and the relative humidity (RH 60–70%) of the rooms where the containers were placed greatly slow down the processes of evaporation and gas transfer during aging.

This suggests that the type of container contributes to chemical differences, but to a lesser extent than the storage time.

The OTHs were reported (Table S4) and OAvs of the main compounds were calculated (Figure S3). The processing data showed that among the different containers, only a small variation occurred in the OAV values, confirming the minor differences among the wines aged in this study.

## 4. Conclusions

The results of this study highlighted the importance of the aging time toward the container type. The aging time, 6 or 12 months, and the type of tank (SS, C, P, or OB) had a significant impact on the composition of volatile compounds in Carignano wine, affecting the aromatic composition of the eight chemical classes identified.

An increase in VOCs was measured from wine at 0_m to 6m, which doubled at 12_m. Plastic vats had the highest increase, followed by concrete and stainless steel, whereas oak barrels had a decrease in the total concentration of VOCs. The most represented chemical class was the alcohol fraction, which decreased during aging; conversely, the ester group increased in the series oak > plastics = concrete > stainless steel.

The compounds influencing the OB_12m samples more were 1-butanol, 2-methyl, lactic acid ethylester, 1-hexanol, and maple lactone.

PCA analysis allowed grouping the samples at the end of the aging period (12 months) regardless of the container, even if minimal clusters along PC2 were evidenced with OB in the upper-left quadrant and P samples in the lower-left quadrant of the graph. The asymmetric distribution of compounds in the loading plot, with 29% located on the right side and 71% on the left side of the multivariate space, determined the separation between the T_6m and T_12m samples observed in the score plot.

Multiple t-test comparisons and two-way ANOVA enabled us to distinguish the effects of the time and container type on the concentration of the different chemical classes analyzed, while also highlighting significant interactions between these two factors.

The relative importance of each factor on the total observed variability through the eta squared (η^2^) showed that the primary factor driving the evolution of the different chemical classes was the time of aging. This was also evidenced by the heatmap of the main single-chemical compounds with a higher abundance of complex compounds at 12_m (esters and lactones).

The trials allowed differentiating among containers and over the aging time; however, the little differences among the various containers in the total VOCs and single-chemical classes can be explained by the containers and environmental characteristics applied.

## Figures and Tables

**Table 1:** Levels (mean ± RSD%) of the sum of chemical classes (aldehydes, acids, alcohols, esters, ketones, and lactones) at 0m, T_6m, and T_12m of aging in different containers.

	Aldehydes	Acids	Alcohols	Esters	Ketones	Lactones
	Mean ± RSD%					
0_m	0.5 ± 1.2	16.1 ± 5.6	70.8 ± 2.1	9.0 ± 2.6	0.8 ± 12.6	0.2 ± 32.3
SS_6m *	0.3 ± 13.4	12.4 ± 6.7	76.4 ± 1.5	7.1 ± 3.8	1.3 ± 15.0	0.2 ± 37.0
SS_12m	0.2 ± 3.3	19.1 ± 4.2	66.4 ± 1.9	8.0 ± 4.2	3.3 ± 8.5	0.3 ± 1.7
C_6m	0.3 ± 7.6	14.2 ± 4.8	76.3 ± 0.3	6.6 ± 4.9	0.7 ± 5.5	0.1 ± 13.5
C_12m	0.2 ± 4.3	16.3 ± 2.9	70.0 ± 0.6	8.4 ± 1.5	1.6 ± 2.6	0.5 ± 11.1
P_6m	0.4 ± 6.2	14.6 ± 5.0	74.8 ± 1.6	8.2 ± 6.9	0.7 ± 8.0	0.1 ± 7.7
P_12m	0.2 ± 9.0	22.0 ± 5.5	61.6 ± 2.0	8.4 ± 2.7	5.0 ± 9.9	0.4 ± 4.9
OB_6m	0.3 ± 15.4	13.7 ± 2.6	76.3 ± 1.5	7.6 ± 6.9	0.8 ± 9.0	0.1 ± 10.7
OB_12m	0.2 ± 6.3	20.0 ± 3.4	66.4 ± 0.7	9.4 ± 2.2	1.4 ± 6.6	0.3 ± 7.2

* SS (stainless steel), C (concrete), P (plastic), and OB (oak barrels); 0_m bulk wine, 6m six months, 12m twelve months.

## Data Availability

The original contributions presented in this study are included in the article/Supplementary Materials. Further inquiries can be directed to the corresponding author.

## Supplementary Materials

The following supporting information can be downloaded at: https://www.mdpi.com/article/10.3390/foods14132290/s1, Figure S1: GC-FID analytical determination of the volatile fraction of Carignano wine after 12 months of aging in (a) stainless-steel tank, (b) plastic vats, (c) concrete vats, and (d) 225L barrique; Figure S2: absolute concentration of ethanol and glycerol in the volatile fraction of the wine aged in the different containers; Figure S3: heatmap of OAVs of the main volatile compounds in the studied Carignano wines at 12 months of aging; Table S1: volatile compounds of Carignano wine after fermentation and during aging in different containers; Table S2: (A) Statistical analysis of different chemical classes over time and across containers. Mean differences were assessed using multiple *t*-tests (0 months vs. 6 months; 0 months vs. 12 months); (B) two-way ANOVA with Tukey’s post-hoc test. Evaluation of the effects of time (6 months vs. 12 months), container type, and their interaction (time × container); Table S3: results of the analysis of variance (ANOVA), sum of squares (SS), and effect sizes (η^2^) for time (6 vs. 12 months), container type, and the interaction “time × container” on different chemical classes; Table S4: odor threshold (OTH) of the main volatile compounds in the studied Carignano wines.
