# Supplementary material for: Influence of Aging Technologies on the Volatile Profile Composition of Carignano cv Red Wines in Sardinia
Source: Foods. 2025 Jun 27;14(13):2290. doi: 10.3390/foods14132290 (PMC12249370; doi:10.3390/foods14132290)
Supplement: Supplementary file 1 [file foods-14-02290-s001.zip › foods-3709184-figures.pdf]

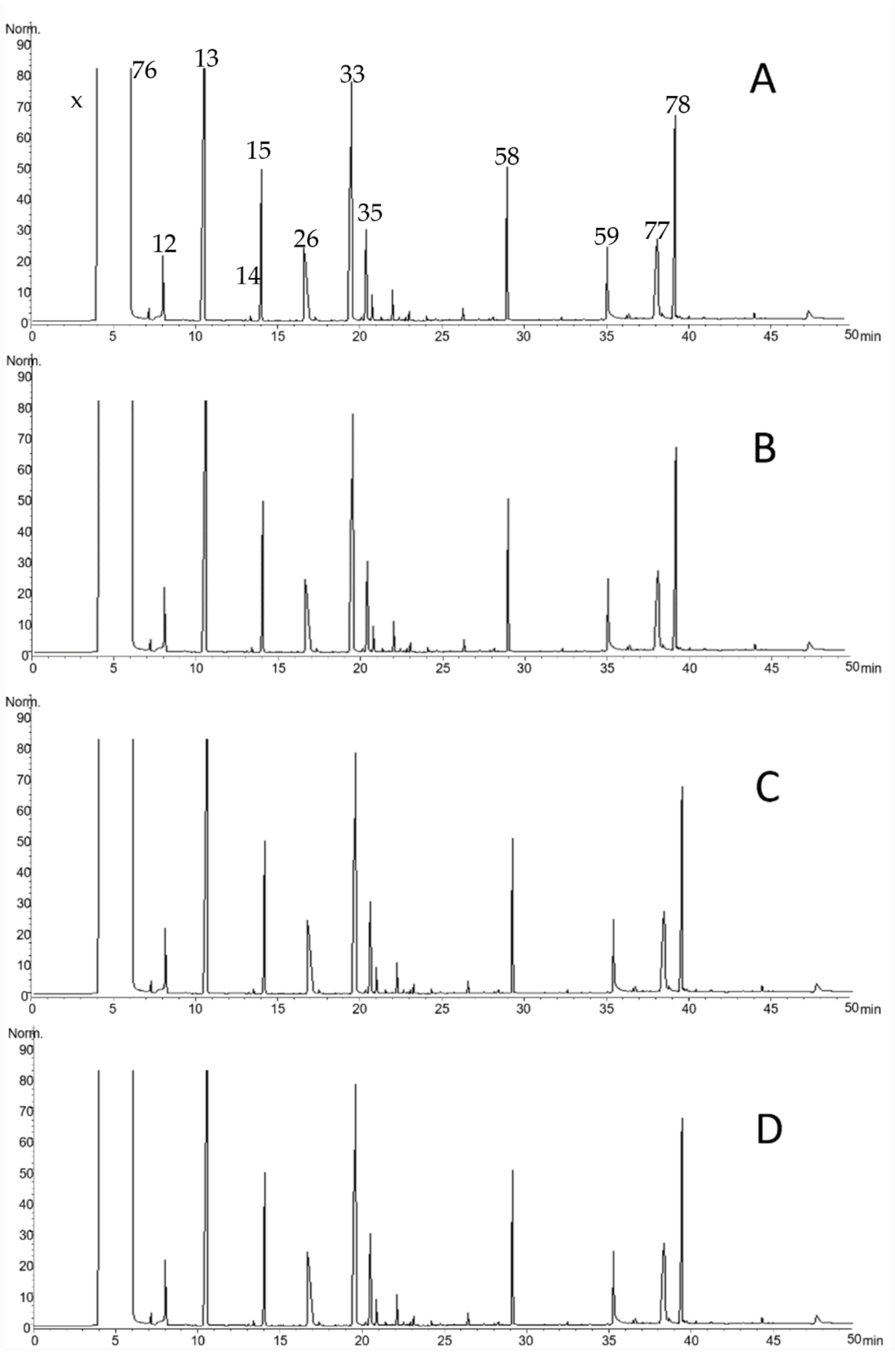

Figure S1. GC-FID analytical determination of the volatile fraction of Carignano wine after 1 year of aging in a) stainless steel tank, b) plastic vats, c) concrete tank, and d) 225L barrique.

X is methanol used as a solvent; the numbers on the peaks refer to the compounds in Table S1.

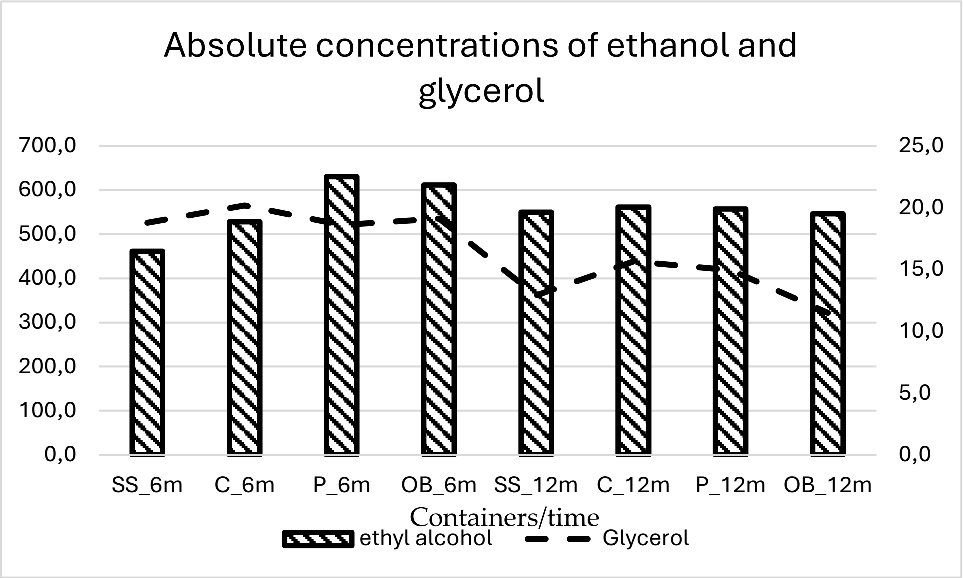

Figure S2. Absolute concentration of ethanol and glycerol in the volatile fraction of the wine aged in the different containers.

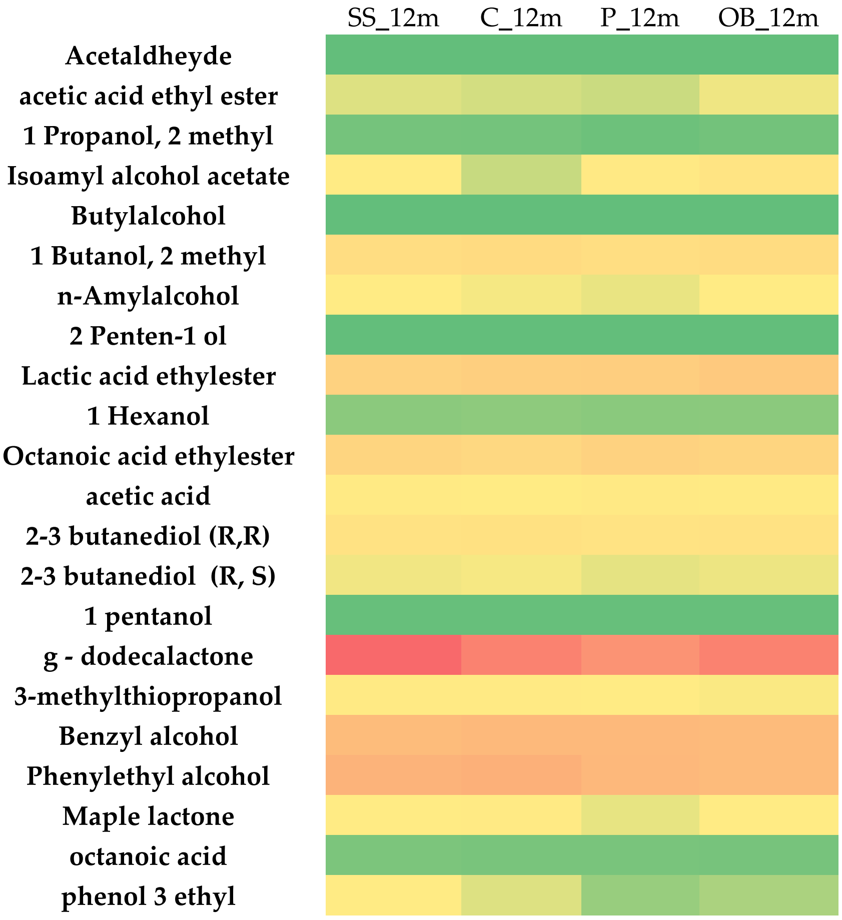

Figure S3. Heatmap of OAVs of the main volatile compounds in the studied Carignano wines at 12 months of ageing.
