# Supplementary material for: Influence of Aging Technologies on the Volatile Profile Composition of Carignano cv Red Wines in Sardinia
Source: Foods. 2025 Jun 27;14(13):2290. doi: 10.3390/foods14132290 (PMC12249370; doi:10.3390/foods14132290)
Supplement: Supplementary file 1 [file foods-14-02290-s001.zip › foods-3709184-tables.pdf]

Table S1. Volatile compounds of Carignano wine after fermentation and during ageing in different containers.

			R.T ± RSD%	start	Stainless steel		Concrete		Plastic		Oak barrels	
	PCA	Sigma Aldrich		% ± RSD%								
	number	number		0_m	SS_6m	SS_12m	C_6m	C_12m	P_6m	P_12m	OB_6m	OB_12m
**Alcohols**												
Ethyl alcohol	76		8.99±6.5	86.76±1.3	81.90±0.6	31.31±5.8	80.53±0.8	29.67±4.2	83.89±1.3	28.32±5.7	81.31±1.8	34.64±1.6
1 Propanol, 2 methyl	10	270466	11.45±8.6	0.24±11.2	0.26±7.2	0.14±3.64	0.29±4.09	0.14±8.8	0.31±6.7	0.09±12.8	0.27±7.1	0.12±9.1
Butylalcohol	12	BX1780	13.14±9.0	0.01±10.3	0.01±8.5	0.01±7.24	0.01±10.1	0.02±10.2	0.01±3.4	0.02±2.8	0.01±13.6	0.01±17.8
1 Butanol, 2 methyl	13	133051	15.17±8.8	0.39±12.5	0.56±14.4	7.47±4.67	0.66±10.7	8.16±3.7	0.61±4.0	7.46±6.7	0.60±5.8	7.67±3.6
n-Amylalcohol	14	W205605	18.22±0.2	1.14±8.8	1.90±7.9		2.30±7.3		2.14±5.1		2.27±12.4	
2 Penten-1 ol	15	304182	18.98±0.1			0.01±13.3		0.00±18.9		0.00±7.5		0.00±11.9
1 Pentanol 3 methyl	20	W376205	21.48±6.8			0.00±9.4		0.00±11.6		0.00±5.3	0.00±7.6	0.00±15.8
1 Hexanol	22	538051	22.75±8.4			0.06±4.7	0.00±9.2	0.07±5.7		0.06±6.9	0.01±13.1	0.06±3.8
2-3 butanediol (R,R)	33	B84904	28.60±7.2	1.36±1.0	2.75±5.1	13.67±3.9	3.37±5.9	14.65±2.7	2.5±13.2	13.58±2.4	3.54±13.0	13.44±1.5
2-3 butanediol (R, S)	35		29.94±7.1	0.59±3.1	1.09±8.8	3.99±5.4	1.09±7.9	4.15±2.3	0.76±9.0	3.89±2.6	1.12±9.9	3.76±0.1
1-2 propanediol	36	471674	30.49±7.3	0.06±9.6	0.09±5.3	0.47±8.2	0.12±14.9	0.47±4.3	0.08±18.6	0.44±4.1	0.12±12.4	0.42±1.9
1 Pentanol	41	138975	32.95±6.5	0.02±8.4	0.01±9.7	0.06±2.8	0.01±13.3	0.05±3.4	0.01±3.0	0.06±16.0	0.01±14.5	0.05±14.9
2-Furanmethanol	42	FLUH99C75 9C8	33.21±0.0			0.01±5.9				0.01±7.1		0.04±3.1
3-Methylthiopropanol	47	W341509	35.35±6.3	0.02±12.0	0.03±7.9	0.11±7.8	0.02±5.9	0.11±3.6	0.02±13.2	0.10±6.7	0.02±8.9	0.08±0.6
Benzyl alcohol α-ethyl	57		41.08±0.0			0.01±4.1		0.02±14.6		0.01±9.4		0.01±5.6
Benzyl alcohol	58	108006	41.21±5.4	0.01±10.3	0.01±13.4	0.07±6.9	0.02±10.1	0.07±2.4	0.01±12.4	0.08±6.6	0.02±6.8	0.07±5.8
Phenylethyl alcohol	59	05512	42.46±5.3	1.55±12.4	2.28±10.1	5.59±6.7	1.96±11.2	5.83±2.0	1.60±4.5	5.44±5.1	1.81±15.0	4.66±2.7
Glycerol	77	G7893	55.62±4.9	3.66±13.2	3.30±4.7	7.34±4.9	3.11±8.3	8.20±19.6	2.47±11.6	7.59±2.9	2.55±15.1	7.28±6.2
diol MW 174*	78		57.15±4.6	1.96±18.0	3.09±10.6	12.65±7.1	3.43±8.4	13.08±5.9	2.77±4.1	12.29±4.9	3.28±13.9	11.26±3.9
**Aldheyds**												
Acetaldheyde	1	402788	4.99±0.7	0.01±3.4	0.01±13.4	< 0.01	0.01±14.4	< 0.01	0.01±5.50	< 0.01	0.01±11.1	< 0.01
Glyceraldehyde	8	49800	10.91±7.8	0.02±5.4	0.03±8.2	0.02±8.73	0.02±14.8	0.01±9.4	0.03±3.7	0.01±9.7	0.02±8.9	0.02±10.1
Furfural	25	185914	23.95±0.1			0.01±11.6		0.01±9.6		0.01±12.2		0.01±14.7
3-Furaldehyde	28		25.52±0.0			0.04±2.5		0.04±3.8		0.04±10.5		0.05±8.6
Benzaldehyde	31	B1334	27.96±0.0			0.01±14.3		0.01±9.4		0.00±13.9		0.01±12.1
Acrolein	62		48.53±0.0			0.01±7.9		0.01±10.7		0.01±18.7		0.01±11.7
**Esters**												
Acetic acid methylester	3		6.16±0.6	0.01±10.4	0.01±8.0		0.01±17.5		0.05±11.4		0.01±13.2	
Acetic acid ethylester	4		7.15±0.3	0.37±7.8	0.26±7.6		0.3±14.5		0.41±11.0		0.35±6.4	
Acetic acid vinylester	6		9.43±0.6		0.00±16.3		0.00±15.9		0.005±4.4		0.00±6.2	
Isoamyl acetate	11	W205508	12.60±5.0	0.00±5.5			0.00±9.7		0.00±9.0	0.01±15.8	0.01±13.3	0.01±7.4
Lactic acid methylester	19		21.35±0.1			0.02±12.3		0.02±16.3		0.02±16.9		0.03±17.7
Lactic acid ethylester	21		22.43±7.9	0.06±11.7	0.23±9.9	2.74±3.6	0.26±10.5	3.00±6.0	0.15±19.2	3.28±7.8	0.26±15.7	3.43±2.3
Octanoic acid ethylester	23		23.21±0.1			0.01±4.2		0.01±7.3		0.01±11.9		0.01±6.9
4 methyl 2 penthyl acetate	27		25.35±0.0							0.01±12.6		0.01±5.4
Unknown ester	30		27.60±0.1			0.02±9.4		0.02±5.8		0.02±7.9		0.02±12.7
Unknown ester	37		31.31±0.0			0.03±7.5		0.01±5.7		0.03±7.9		
Butanoic acid 2-methyl ester	43		33.57±0.1			0.08±3.7		0.08±2.4		0.07±5.8		0.06±2.6
Methyltriglicolacetate	46		34.50±0.1						0.04±8.3		0.04±18.1	
Trimethylene acetate	50		35.99±0.0			0.00±10.3		0.00±18.5				0.00±7.3
undecanoic acid metilester	51		36.06±6.2	0.01±10.2	0.01±15.2	0.04±8.3	0.01±6.2	0.05±1.4	0.01±10.5	0.04±14.1	0.01±17.1	0.04±2.0
Acetic acid diethyl ester	52		38.55±5.6	0.18±13.9	0.24±11.2	0.47±6.0	0.24±10.2	0.48±8.7	0.19±9.9	0.47±6.1	0.24±9.5	0.36±2.9
Unknown ester	53		39.15±0.0			0.01±5.9		0.01±9.7		0.01±8.7		
Unknown ester	54		39.72±4.8		0.02±7.9	0.01±12.1	0.01±18.3	0.01±8.6		0.01±4.1	0.02±9.3	0.01±6.9
Unknown ester	56		40.65±0.0			0.02±9.3		0.02±15.8		0.02±11.2		0.01±6.8
Glycerol α monoacetate	70		54.25±0.0			0.07±6.4		0.08±10.9		0.08±13.3		0.07±7.0
Unknown ester	72		56.14±0.0			0.15±2.9		0.16±6.9		0.13±12.7		0.13±12.4
**ketones**												

2-Butanone	5	360473	7.50±0.1		0.01±10.9		0.01±6.4		0.01±6.7		0.02±8.9	
2-Butanone 3 Hydroxy	17	8.20664	20.33±8.6	0.05±12.5	0.14±13.2	1.60±7.2	0.08±5.3	0.76±4.9	0.06±14.6	2.51±8.0	0.08±8.8	0.65±7.9
2-Propanone 1 Hydroxy	18		20.98±0.1			0.03±9.2		0.03±11.8		0.07±11.6		0.04±15.5
**Acids**												
Butanedioic acid 2, 3-dihydroxy	2		5.85±1.0	0.00±2.2	0.00±12.1				0.00±11.5			
Nonaoic acid	7	N5502	10.26±0.5		0.01±10.7		0.04±10.2		0.06±10.3		0.01±19.4	
Acetic acid	26	A6283	24.53±8.0	1.08±8.5	1.25±2.7	8.39±3.6	1.63±4.9	6.75±4.4	1.36±8.5	9.53±6.1	1.58±8.6	7.72±4.3
Propanoic acid 2-methyl	34		29.62±0.0			0.06±3.7		0.06±2.5		0.06±3.6		0.05±4.6
Butanoic acid	38		31.98±0.0			0.02±7.3		0.02±3.6		0.01±11.8		0.01±9.0
Sorbic acid	48	S1626	35.58±0.0			0.00±14.1		0.00±18.0		0.00±12.6		0.00±8.9
Caproic acid	55	153745	40.00±5.3	0.01±13.8	0.01±14.0	0.04±14.4	0.01±3.1	0.05±10.8	0.02±10.3	0.05±6.5	0.02±13.7	0.03±14.6
Octanoic acid	61	C2875	47.25±4.5	0.01±9.5	0.03±13.9	0.05±5.9	0.02±14.2	0.04±7.7	0.03±15.8	0.04±17.3	0.02±15.2	0.04±12.3
Lactic acid	67	W261106	51.29±0.1			1.05±6.6		1.34±10.8		1.90±7.1		1.70±4.2
**Lactones**												
γ - Dodecalctone	45	W240109	34.20±0.0			0.01±6.4		0.00±12.8		0.00±14.9		0.00±11.9
d-Heptalactone	49	W253901	35.84±0.0			0.01±14.7		0.01±11.5		0.02±3.4		0.01±16.9
Maple lactone	60		45.26±2.3			0.04±5.5		0.05±10.6		0.03±10.5	0.01±6.3	0.04±10.9
Heptanolactone	65		50.67±0.0			0.03±6.0		0.04±8.5		0.04±7.5		0.04±7.9
Nonalactone	73		57.39±0.0			0.05±4.9		0.14±16.9		0.15±11.2		0.04±5.6
**Ethers**												
Di-tert-butoxy-methane	29		26.86±0.0			0.02±12.5		0.01±11.3		0.02±2.8		0.02±11.5
Unknown Ether	63		49.29±5.7			0.02±15.5		0.02±1.3		0.01±6.2		0.02±13.3
**Others**												
Undecane	9	U407	11.23±5.3		0.00±11.9	0.05±7.9	0.00±10.9	0.05±12.5	0.00±10.0		0.00±7.8	
Thiosemicarbazide	16	T33405	19.77±0.2	0.00±11.3	0.00±17.8		0.00±9.4		0.00±9.7		0.00±6.5	
1,3 Butadiene 2,3-dimethyl	24	145491	23.61±0.6							0.00±16.7		0.00±15.6
Hydrocarbon	32		28.17±0.0			0.00±11.7		0.00±8.7				
Ethane isothiocyanate	39		32.22±0.0			0.12±10.8		0.13±9.1		0.12±12.3		0.10±4.7
Unknown	40		32.36±7.2		0.03±7.7	0.31±6.9	0.03±9.8	0.33±3.3	0.02±13.4	0.31±3.4	0.02±10.9	0.29±1.6
Quinoline	44	94517	33.80±5.7	0.08±13.5	0.15±9.1	0.51±2.8	0.14±16.0	0.53±3.9	0.04±9.4	0.51±2.1	0.07±3.6	0.57±1.8
Unknown	64		50.45±0.0			0.02±2.7		0.04±10.9		0.03±2.4		0.02±8.5
Phenol 3 ethyl	66		51.08±4.8	0.05±5.8		0.01±7.7		0.01±7.9				
Butanedioic anidride	68		52.70±0.0			0.02±2.5		0.02±9.1		0.02±10.8		0.02±17.6
N-Hexyl carbytol	69		52.99±0.0			0.09±10.8		0.09±10.3		0.11±6.9		0.08±3.3
Unknown	71		55.87±0.1			0.15±5.8		0.16±8.4		0.16±12.4		0.14±1.8
Propylbenzene	74	82119	57.61±0.0			0.04±5.9		0.07±14.8		0.07±14.6		
Unknown	75		58.50±0.0			0.06±4.8		0.06±9.72		0.05±17.8		0.09±1.7

Table S2A. Statistical analysis of different chemical classes over time and across containers. Mean differences were assessed using multiple t-tests (0 months vs 6 months; 0 months vs 12 months).

**"*p*" Differences over time (0m *vs* 6m and 0m *vs* 12m)**						
	Aldehydes	Acids	Alcohols	Esters	Ketones	Lactones
0_m *vs* SS_6m	**0.0412**	**0.0270**	**0.0262**	**0.0031**	0.0664	>0.9999
0_m *vs* SS_12m	**< 0.0001**	**0.0420**	0.0700	**0.0383**	**0.0005**	0.1531
0_m *vs* C_6m	**0.0004**	0.1710	**0.0132**	**0.0019**	0.6267	0.4805
0_m *vs* C_12m	**< 0.0001**	>0.9999	>0.9999	0.0527	**0.0007**	**0.0109**
0_m *vs* P_6m	**0.0130**	0.3655	0.0902	0.2954	0.8361	0.5097
0_m *vs* P_12m	**< 0.0001**	**0.0113**	**0.0048**	0.1404	**0.0005**	**0.0074**
0_m *vs* OB_6m	**0.0120**	0.0500	**0.0284**	**0.0475**	>0.9999	>0.9999
0_m *vs* OB_12m	**< 0.0001**	**0.0230**	**0.0345**	0.6149	**0.0046**	0.2371

Table S2B. Two-way ANOVA with Tukey's post-hoc test. Evaluation of the effects of time (6 months vs 12 months), container type, and their interaction (time × container).

	***"p"* Differences over time (6m *vs* 12m) and container**					
	Aldehydes	Acids	Alcohols	Esters	Ketones	Lactones
SS_6m *vs* SS_12m	**<0.0001**	**<0.0001**	**<0.0001**	0.1645	**<0.0001**	**0.0014**
SS_6m *vs* C_6m	0.0600	0.1600	>0.9999	0.6450	0.0553	0.7222
SS_6m *vs* C_12m	**<0.0001**	**0.0002**	**<0.0001**	**0.0092**	0.5894	**<0.0001**
SS_6m *vs* P_6m	0.7400	**0.0452**	0.5023	**0.0354**	0.0604	0.7928
SS_6m *vs* P_12m	**<0.0001**	**<0.0001**	**<0.0001**	**0.0063**	**<0.0001**	**<0.0001**
SS_6m *vs* OB_6m	0.3726	0.5172	>0.9999	0.7601	0.2311	0.9955
SS_6m *vs* OB_12m	**<0.0001**	**<0.0001**	**<0.0001**	**<0.0001**	0.9787	**0.0031**
SS_12m *vs* C_6m	**0.0094**	**<0.0001**	**<0.0001**	**0.0059**	**<0.0001**	**<0.0001**
SS_12m *vs* C_12m	>0.9999	**0.0075**	**0.0064**	0.7718	**<0.0001**	**<0.0001**
SS_12m *vs* P_6m	**<0.0001**	**<0.0001**	**<0.0001**	0.9867	**<0.0001**	**<0.0001**
SS_12m *vs* P_12m	0.9310	**0.0087**	**0.0004**	0.6619	**<0.0001**	**0.0002**
SS_12m *vs* OB_6m	**0.0011**	**<0.0001**	**<0.0001**	0.9132	**<0.0001**	**0.0004**
SS_12m *vs* OB_12m	>0.9999	0.9856	>0.9999	**0.0031**	**<0.0001**	0.9999
C_6m *vs* C_12m	**0.0097**	**0.0450**	**<0.0001**	**0.0003**	**0.0015**	**<0.0001**
C_6m *vs* P_6m	**0.0026**	0.9956	0.5799	**0.0011**	>0.9999	>0.9999
C_6m *vs* P_12m	**0.0010**	**<0.0001**	**<0.0001**	**0.0002**	**<0.0001**	**<0.0001**
C_6m *vs* OB_6m	0.9429	0.9891	>0.9999	0.0624	0.9888	0.9780
C_6m *vs* OB_12m	**0.0184**	**<0.0001**	**<0.0001**	**<0.0001**	**0.0093**	**0.0001**
C_12m *vs* P_6m	**<0.0001**	**0.1600**	**0.0003**	0.9960	**0.0017**	**<0.0001**
C_12m *vs* P_12m	0.9267	**<0.0001**	**<0.0001**	>0.9999	**<0.0001**	0.7584
C_12m *vs* OB_6m	**0.0011**	**0.0092**	**<0.0001**	0.1710	**0.0075**	**<0.0001**
C_12m *vs* OB_12m	>0.9999	**0.0014**	**0.0066**	0.0620	0.9768	**<0.0001**
P_6m *vs* P_12m	**<0.0001**	**<0.0001**	**<0.0001**	0.9826	**<0.0001**	**<0.0001**
P_6m *vs* OB_6m	0.0231	0.7833	0.6022	0.4684	0.9921	0.9906
P_6m *vs* OB_12m	**<0.0001**	**<0.0001**	**<0.0001**	**0.0163**	**0.0102**	**0.0002**
P_12m *vs* OB_6m	**0.0001**	**<0.0001**	**<0.0001**	0.1230	**<0.0001**	**<0.0001**
P_12m *vs* OB_12m	0.7930	0.0460	**0.0004**	0.0880	**<0.0001**	**<0.0001**
OB_6m *vs* OB_12m	**0.0021**	**<0.0001**	**<0.0001**	**0.0003**	**0.0460**	**0.0008**

Table S3. Results of the analysis of variance (ANOVA), sum of squares (SS), and effect sizes (η^2), for time (6 *vs* 12 months), container type, and the interaction "time × container" on different chemical classes.

Effect size index (time, 6m *vs* 12m, and container)				
	Source	Sum of square	*p*-value	(η^2) eta-squared
	A (time)	0.1425	< 0.0001	0.79
	B (container)	0.0070	**0.060**	
**Aldehydes**	A *x* B (interaction)	0.0174	0.002	0.07
	Residual	0.0120		
	Total	0.1789		
	A (time)	180.0	< 0.0001	0.74
	B (container)	29.75	< 0.0001	0.12
**Acids**	A *x* B (interaction)	23.03	0.0001	0.09
	Residual	9.110		
	Total	242.0		
	A (time)	590.0	< 0.0001	0.13
	B (container)	75.33	< 0.0001	0.10
**Alcohols**	A *x* B (interaction)	35.30	0.0002	0.05
	Residual	15.01		
	Total	715.6		
	A (time)	7.921	< 0.0001	0.46
	B (container)	4.640	0.0002	0.27
**Esters**	A *x* B (interaction)	2.510	0.0040	0.15
	Residual	1.999		
	Total	17.070		
	A (time)	22.29	< 0.0001	0.47
	B (container)	12.40	< 0.0001	0.26
**Ketones**	A *x* B (interaction)	11.76	< 0.0001	0.25
	Residual	0.726		
	Total	47.18		
	A (time)	0.3723	< 0.0001	0.74
	B (containaer)	0.0393	0.0001	0.08
**Lactones**	A *x* B (interaction)	0.0744	< 0.0001	0.15
	Residual	0.0155		
	Total	0.5015		

Table S4. Odor threshold (OTH) of the main volatile compounds in the studied Carignano wines.

	OTH	Descriptor
Alcohol		
1 Propanol, 2 methyl	40	Alcohol, ripe fruit, pungent, harsh
Butylalcohol	150	Alcoholic odor
1 Butanol, 2 methyl	60	Herbaceous, whiskey, malt, burnt
n-Amylalcohol	60	Bouquet, astringent
2 Penten-1 ol	64	ethereal, fruity odor cherry
1 Hexanol	8	Light branches, leaves and fruity odor
2-3 butanediol (R,R)	150	Like rubber chemical odor
2-3 butanediol (R, S)	150	Like rubber chemical odor
1 Pentanol	60	Bouquet, astringent
3-Methylthiopropanol	3	Raw potatoes odor, alliaceous odor
Benzyl alcohol	0.2	Bitter apricot seed odor
Phenylethyl alcohol	14	sweet rose odor
Acetaldheyde	100	Bitter almond
Acetic acid ethylester	12	Fruity odor, ester odor
Isoamyl acetate	0.16	Banana
Lactic acid ethylester	14	Butter, cream, fruit
Octanoic acid ethylester	0.005	Fruity odor, fennel odor, sweet odor
Acetic acid	200	Vinegar
Octanoic acid	10	Sweat, cheese, oily, fatty, rancid, soapy, sweet, faint fruity, butter
γ - Dodecalactone	0.007	
Maple lactone	1.2	Caramell, licorice
Phenol 3 ethyl	0.44	Smoky, phenolic, barnyard
